# Notes and News

**Published:** 1889-11-30

**Authors:** 


### Notes and News.

Collected and Communicated.

The annual report of the Birkenhead Borough Hospital contains a handsome list of donations resulting from various pastimes. The proceeds of a ball are down at £243, receipts for admission to view ss. "City of New York," £75; proceeds of children's fairy play, £23; and concert at Kensington House School, £20. Thus can those who play help those who are in pain.

The fourth series of the successful subscription dances, called the Portman Cinderellas, given in aid of the Great Northern Central Hospital, Holloway-road, will be held on Thurdays, December 19, January 9 and 23, and February 6 and 20. Tickets are obtainable only upon introduction by a patroness or steward. Prospectuses, containing all information, will be sent on application to the secretary of the Great Northern Central Hospital, Holloway-road, N. Amongst the stewards are Lord Charles Beresford, Dr. Burnet, Dr. Dakin, and Colonel Milman.

In consequence of complaints as to the large number of foreign doctors practising in France, the Minister of Public Instruction has just decided that permissions to practise shall only be given after a searching enquiry as to the qualifications of the candidates. Until within the last two years no great difficulty was experienced in obtaining this permission, although it does not appear to be necessary, however, as some *forty* practitioners are said by the *Standard* to be practicing there without any sort of authorisation or any French degree. Dr. Lutaud states that the prefect, in whose hands the matter will rest, will refrain from disturbing these practitioners. Paris is, however, supplied with a great number of English and American medical men who hold a French qualification, and no others will be allowed to practice there unless similarly qualified.

We ought to have noticed before the report of the annual meeting of the Royal Seamen's and Marines' Orphan School which was sent us, but we hope it is not yet too late to mend. The report was in every way satisfactory. In the course of an excellent speech, Admiral Sir E. Commerell said it was a crying shame that they got such little support in Portsmouth. He did not believe it was because there was any want of sympathy with the sailor or the marine, but he thought they did not, in the large towns, know what a charity they had here, and how admirably it was conducted. A great many people did not subscribe to charities because the money often went, not in charitable purposes, but to all sorts of things around the charity. But here they educated excellently well and religiously 159 girls and 63 boys. First of all they had a religious education, which was a great point, and then they had an education that fitted them for a very difficult thing nowadays—to be good servants.—The children had their annual treat the following day.

Another noble gift, and this time Sir Edward Guinness is the giver. Whatever may be said of the blots on our modern civilisation, it cannot be said that efforts are not made to lessen them. The housing of the poor is a subject which sentimental ladies, platform orators, and grumblers in general are always moaning or ranting about, but Sir Edward Guinness, who belongs to none of the above classes, is the person who comes forward with £250,000 carefully placed in trust to aid the cause of better housing. The gift is generous, and excellent arrangements are made to prevent its being misapplied.

The Manchester Hospital for Incurables is a magnificent building at Mauldeth, far away from the smoke of the great town. At Walmersley, too, thanks to the munificence of a generous lady, the committee of the hospital have now a second home in another healthy and breezy district, and at both of these well-conducted establishments by pleasant and varied recreation, by watchful oversight, and by unremitting care, the lives of the inmates are made as happy as may be. The Bishop of Manchester, as chairman, has been calling attention to the good done by these homes, but pleads for more funds, so that more cancer cases can be taken.

The monthly meeting of the Board of Management of the Manchester Royal Infirmary was held in that institution on Monday, Mr. J. W. Maclure, M.P., presiding. Mr. Joseph Collier, M.B., F.R.C.S., London, was appointed surgical registrar to the infirmary, in the place of Dr. William Thorburn, who has become an assistant surgeon. Mr. W. H. Iddon, M.B., London, was chosen assistant medical officer in the stead of Mr. Joseph Clegg, who has resigned. Mr. A. W. W. Lea, M.R.C.S., L.R.C.P, London, was appointed house surgeon, and Mr. Alexander Wilson, M.R.C.P., L.R.C.P., was re-elected administrator of anæsthetics.

There has at last been a calm and business-like meeting of the governors of Suffolk General Hospital. The chairman, the Duke of Grafton, made a pacific speech, and the following resolution was passed: "That in consequence of the decision arrived at on the 15th October, this meeting rescinds rules 9 and 17 as revised, and that a special meeting be summoned for the 11th February, 1890, at one o'clock." The Duke thought that if the meeting was called for the 11th February, a month before the annual meeting, the subject could be thoroughly discussed in the hope of avoiding a heated discussion, similar to those which have occurred before. It really looks as though the storm were blowing over, and that the New Year may see this disgraceful quarrel at an end.

The care of inebriates is often regarded as most hopeless and trying work, and many regard the attempt as all but Quixotic. There are, however, certain sisters from St. Raphael's, Woodside, Croydon, who, when begging in their irresistible manner, say: "Then there is our Home for Women Inebriates!" It needs a bold man to reply that he doesn't believe much good can be effected by that home, for statistics are poured forth, and examples quoted, which bear every investigation. They say that, beyond all question, a great number of inebriates can be cured, and certainly one-half; and very likely three out of every four patients, if the friends of the patients were not so ready to believe that great improvement is the same thing as perfect recovery, and would consent to leave them under treatment for longer periods; but perhaps one-fourth are chronic invalids, requiring the same attention and treatment as patients suffering from some incurable disease. The home is beautifully situated in eighteen acres of ground, and we can quite believe that if cures are effected anywhere, they are at St. Raphael's.

THE following vacancies are announced this week, the amount of the salary in each case being given after the name of the institution :

**Resident Medical Officers.**

Carmarthen Asylum. Junior Assistant Medical Officer—£100, board, &c.
Leeds Borough Hospital. Resident Medical Officer—£150, board, &c.
Wimbledon Convalescent Hospital. Resident Medical Officer and Superintendent—£150, board, &c.
Wolverhampton Hospital. Resident Assistant—Board, &c.
Pinxton Collieries. Resident Surgeon—£300, house, &c.
Monkstown Hospital. Resident Medical Officer—£40, board, &c.

**Non-Resident Medical Officers.**

Mullingar Union. Medical Officer of Health—£145.
West End Hospital, Welbeck-street. Surgeon.
North-West London Hospital. Assistant Physician.
St. George's Hospital. Lecturer on Dental Surgery.

THE Victoria Hospital for Children at Chelsea issues a special appeal for funds, signed by Lord Cadogan. It is a pretty little hospital, always bright and tidy, and excellently managed ; it deserves all the support it asks. There is a convalescent branch at Margate, and unless we are misinformed there is a small pay-wing at Chelsea, where children of the wealthier classes can be taken in for operations or other treatment. The special appeal is prettily illustrated with four children's faces.

THE Head Constable of Liverpool gives statistics of accidents in that town for twelve months. During the year ending September 29, 1889, there were 2,110 accidents, of which 86 were fatal. The police saved 39 lives, and the lives of 265 additional persons were saved by others, illustrating the ready help which bystanders and persons passing render in cases of accident. Of the total number, assistance was rendered by the police in 1,540 cases, or 73 per cent. What a good thing the police are instructed in first aid and ambulance classes.

CANON ALLEN lately preached a special sermon on the anniversary of the dedication of the chapel attached to Ripley Hospital, Lancaster. The institution was founded in memory of the late Mrs. Ripley, and, in speaking of her, Canon Allen said : "She did not devote a large portion of her time in prayers and attendance at church, though she used to come regularly to church on Sundays and twice on week-days—Wednesday and Friday mornings—which, he was sorry to say, few did now." Surely this reference was not in the best taste. Mrs. Ripley would have been the last to have desired her presence in a place of worship noted and commented on. The offertory was in aid of the building fund of the new infirmary.

WE have received a letter from a correspondent in Baltimore, pointing out that our statistics of the long lives of Quakers can be beaten by the statistics issued from Sandy Spring on the Ohio Railway. We are not surprised ; we never told a story or stated a fact yet but what an American promptly "went one better." So we meekly quote a few of the statistics sent us, and retire from the field. "Now there was the Penn family. Mary lived to be 109 years old. Edward died at 104, Lizzie was 103 when she died, and Joseph was 101. Joshua lived to be 99 and 10 months. Mary No. 2 was 98, and another Mary was 89. William Thompson was one of the oldest men in town. He died at 113 years. Isaac Moore lived to be 102. Mrs. Russell died at 104. Mrs. Kirk was 101. Billy Matthews and Will McCormick were each 101 when they died. Billy Simpson was 100, and Mahlon Chandlee is now living at 100. Cornelius Sullivan was 94, William Brown was 92 when he left us, and Jimmy Whiteside is still living, hale and hearty, at 96."

MRS. KEELEY, the veteran actress, celebrated her birthday last week, and amongst her visitors were four little patients from the Hospital for Sick Children, who took a handsome bouquet of flowers to one who has been a most kind friend to them. The Hospital for Sick Children is now trying—and so far with success—to get all the public schools, such as Eton and Harrow, to maintain a cot within its wards.

MR. R. W. Boyd, of the Sanitary Institute, has published a very useful little book, called "The A B C of a Healthy House." In it are plainly and concisely set forth the principal points to be attended to to secure perfect sanitation, and the legal rights of a householder with regard to buildings under the control of the Office of Works. There is an excellent index at the end of the book, so that it is easy to turn at once to any subject desired.

THE cottage hospital at Wirksworth continues to do good work. The receipts for the past year from all sources have been £230 6s. 6d., and the expenditure £205 18s. 10d., and there is a balance in favour of the hospital of 10s. 7d. Last year there was a balance due to the treasurer of £23 17s. 1d. The number of patients has been 48, against 39 in 1888. The medical officers are heartily thanked for their services, which have been most successful, as not a single patient has died in hospital this year.

THE subject of dissection is one which the lay mind can scarcely contemplate calmly. Like the fact that our beef and mutton were once cows and sheep, the less it is talked about the better, but the necessity must be recognised. The Paddington Board of Guardians at their late meeting passed a resolution approving the provisions of the Anatomical Act, and this in spite of the fact that there was a lady on the board. We congratulate the guardians on having acted in accordance with the interests of science, and of the "greatest happiness of the greatest number."

AT the Holborn Town Hall, on Tuesday next, December 3rd, a grand ball will be given in aid of the funds of the Royal Free Hospital, Gray's Inn-road. A local committee of gentlemen connected with the parish of St. Pancras has been engaged for some time past organising this festivity, and the patronage has been secured of Sir Julian Goldsmid, the Hon. Cochrane-Baillie, and Robert Grant Webster, Esq., three of the four parliamentary representatives of the borough. Tickets, and all information, may be obtained of Mr. R. G. Hartley, 8, Derby-street, W.C., the hon. sec. ; or of Mr. J. Balls, treasurer, 362, Gray's Inn-road.

STANLEY does not seem to be able to say enough in praise of Dr. Parke. In the letters printed in the papers this week Stanley says there were all kinds of complaints amongst his men, but the most numerous, and those who gave the most trouble, were those who suffered from ulcers. So largely had these drained the medicine chests that the surgeon had nothing left for their disease but pure carbolic acid and permanganate of potash. Nevertheless, there were some wonderful recoveries during the halt of Stair's column on the Ituri River in January. The surgeon's "devotion" —there is not a fitter word for it—his regular attention to all the minor details of his duties, and his undoubted skill, enabled Stanley to turn out 280 able-bodied men by April 1, sound in vital organs and limbs, and free from all blemish ; whereas on February 1 it would have been difficult to have mustered 200 men in the ranks fit for service. "I do not think I ever met a doctor who so loved his 'cases.' To him they were all 'interesting,' despite the odours emitted, and the painfully qualmish scenes. I consider this expedition in nothing happier than in the possession of an unrivalled physician and surgeon, Dr. F. H. Parke, of the A.M.D."

